# Biochemical profiling of metabolomics in heavy metal-intoxicated impaired metabolism and its amelioration using plant-based bioactive compound

**DOI:** 10.3389/fmolb.2022.1029729

**Published:** 2022-10-18

**Authors:** Azka Yaqoob, Kanwal Rehman, Muhammad Sajid Hamid Akash, Maria Alvi, Syed Muhammad Shoaib

**Affiliations:** ^1^ Department of Pharmaceutical Chemistry, Government College University, Faisalabad, Pakistan; ^2^ Department of Pharmacy, The University Multan, Multan, Pakistan; ^3^ Drugs Testing Laboratory, Faisalabad, Primary & Secondary Healthcare Department, Government of the Punjab, Faisalabad, Pakistan

**Keywords:** lead acetate, quercetin, lipid metabolism, amino acid metabolism, gene expression

## Abstract

Exposure to Pb is widely spreading and has far-reaching negative effects on living systems. This study aimed to investigate the toxic effects of Pb, through biochemical profiling and the ameliorative effects of quercetin against Pb-toxicity. Twenty-five male Wistar albino mice were divided into the following five groups. The CON-group received normal saline; the Pb-group received PbAc; the Pb + Q-CRN group received lead acetate followed by quercetin; the Q-CRN group received quercetin; and the CRN group received corn oil. After 4 weeks, the mice were euthanized. It was speculated that Pb significantly increased the levels of serine, threonine, and asparagine and decreased the levels of valine, lysine, and glutamic acid in the plasma of Pb-group, thus impairing amino acid metabolism. However, in the Pb + Q-CRN group, the level of these six amino acids was restored significantly due to the ameliorative effect of quercetin. The presence of lipid metabolites (L-carnitine, sphinganine, phytosphingosine, and lysophosphatidylcholine) in mice serum was confirmed by ESI/MS. The GPx, SOD, GSH, and CAT levels were significantly decreased, and the MDA level was significantly increased, thus confirming the oxidative stress and lipid peroxidation in the Pb group. The antioxidant effect of quercetin was elucidated in the Pb + Q-CRN group. Expression of CPT-I, CPT-II, LCAT, CROT, CACT, and MTR genes was significantly upregulated in the liver of Pb goup mice. Hence, the findings of this study proved that Pb exposure induced oxidative stress, upregulated gene expression, and impaired the lipid and amino acid metabolism in mice.

## 1 Introduction

Metabolomics is the study of metabolites and metabolism occurring in an organism. Metabolomics can also be defined as the metabolite composition or the metabolomes in the organism, organ, tissue, or cell (Turi et al., 2018). Metabolomics is one of the branches of “omics” science. The omics science has several branches, including genomics, proteomics, transcriptomics, inomics, phenomics, and metabolomics (Chakraborty et al., 2022). The definition of metabolomics is the quantitative and qualitative characterization of smaller molecules that show some changes in response to stimuli from external and internal sources, having a molecular weight of less than 1,000–1,500 Da (Belhaj et al., 2021). From genomics to proteomics, the information provided signifies the occurrence in the cell (that affects phenotype, epigenetic regulation, and post-translational modifications). However, on the other hand, metabolomes help capture the physiology or pathophysiology of the host and its response to the environment (Manzoni et al., 2018). Metabolomics is widely used in different disciplines and is a valuable tool in the investigation of biomarkers (Klein and Shearer, 2016; Zhang et al., 2016), drug discovery (Lu and Chen, 2017; Mercier et al., 2018), conformation of biotransformation pathways (Ren et al., 2016; Zhang et al., 2016), and pathogenesis of diseases (Ren et al., 2016; Würtz et al., 2016). Metabolomics studies not only help in the identification of endogenous substances in biological samples such as blood, urine, etc. but also help explain the differences among different conditions by performing statistical analysis. Toxicology, nutrition, clinical trials, and pharmacology-like fields have already utilized metabolomics studies for different purposes (Brignardello et al., 2017; Korsholm et al., 2017).

Lead (Pb) is a bluish–grey metal present in the crust of the Earth. For many years, Pb has been employed in a variety of industrial, agricultural, and home purposes (Kumar et al., 2020). Nowadays, mankind is more exposed to Pb due to human-induced activities such as oxide synthesis for pigments and paints, burning of fossil fuels, production of lead-acid batteries, mining, and different manufacturing processes (Rehman et al., 2018; Briffa et al., 2020). Some other sources of lead exposure are lead industries, mining, ceramics, petrol pumps, printing press, lead pipes, cosmetics, toys, jewelry, and soil (Qader et al., 2021). The human body is exposed to Pb *via* inhalation and ingestion routes of contaminated food and water. After entering the body, Pb is absorbed through the intestinal route and distributed to different tissues via blood supply. It is accumulated in the soft tissues (brain, liver, spleen, and lungs), bone, and blood in the body (Abd El-Hack et al., 2019). When Pb enters the body, it interacts with proteins and inhibits calcium action, amide, and sulfhydryl enzymes. The main route of its toxicity is oxidative stress in the liver, resulting in the suppression of antioxidant enzymes such as catalase, superoxide dismutase (Chakraborty et al., 2022), glutathione reductase (GR), and glutathione peroxidase (GPx) (Ramah et al., 2019; Rozier and Liebelt, 2019). The GSH molecule has the sulfhydryl group–SH in its structure to whom Pb has a greater affinity (Vacchi-Suzzi et al., 2018; Balali-Mood et al., 2021). Lead increases the level of reactive oxygen species (superoxide (O^2-^), hydroperoxide (−O−O−H), and hydrogen peroxide (H^2^O^2^) and leads to alteration in lipid metabolism, DNA damage, gene expression, membrane integrity, and different physiological processes (Balali-Mood et al., 2021). Lead can also interfere with the synthetic pathway of heme, and thus it is also responsible for anemia (Rehman et al., 2018; Javorac et al., 2021).

**FIGURE 1:**
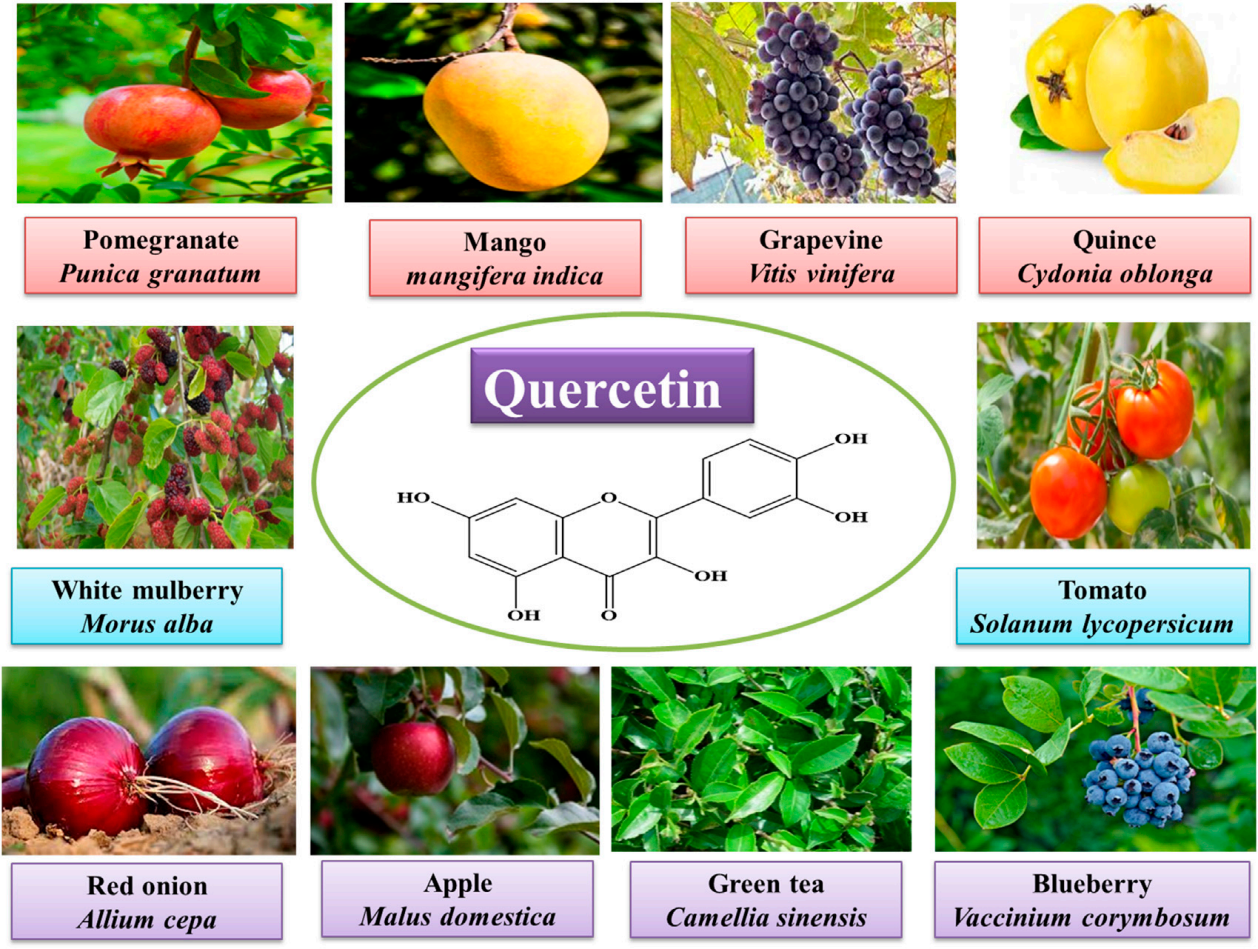
Sources and molecular structure of quercetin.

Quercetin [2-(3,4-dihydroxyphenyl)-3,5,7-trihydroxychromen-4-one] is a bioflavonoid compound that contains many phenol rings. It can be obtained from plants and is also found in many fruits and vegetables (Figure 1). The beneficial effects of quercetin are associated with its structure (Akinmoladun et al., 2020). It is known for its antihypertensive, anti-obesity, anti-atherosclerotic, anti-inflammatory, vasodilatory, and anti-hypercholesterolemia activities. The molecular formula of quercetin is C_15_H_10_O_7_, and its chemical structure is also shown in Figure 1. It has unsaturated and phenolic hydroxyl groups that are responsible for its strong antioxidant activity. The anti-inflammatory and antioxidant properties of quercetin are responsible for its role in the treatment and prevention of cancer and cardiovascular diseases (Yang et al., 2020). Quercetin has strong antioxidant activity due to which it is widely used in traditional Chinese and botanical medicine (Xu et al., 2019). Quercetin, by following different pathways, can prevent the biological system from the damage induced by reactive oxygen species (ROS) (Taşlı et al., 2018). Quercetin’s antioxidant action is primarily demonstrated by its effects on ROS, GSH, signal transduction pathways, and enzymatic activity (Xu et al., 2019). It is also thought that by inhibiting lipid peroxidation, quercetin can prevent several degenerative diseases (Akinmoladun et al., 2020).

This study aims to determine the effect of Pb toxicity on serum metabolomes of lipid and amino acid metabolism in the mouse model. The gene expression of CPT-I, CPT-II, LCAT, CROT, CACT, and MTR has been investigated by qRT-PCR. In this study, after Pb toxicity, the post-treatment with quercetin blended in corn oil helps in determining the ameliorative effect of quercetin against lead toxicity impaired metabolism through biochemical profiling.

## 2 Methodology adopted

### 2.1 Chemicals and assay kits

Lead acetate (PbAc) (Merck & Co., New Jersey, United States), quercetin (BEARS ORGANICS, LLC, Mapleton, Utah, United States), glutathione (GSH) (Catalog Number; E-EL-R2491, Elabscience Biotechnology Inc. Houston, Texas, United States), superoxide dismutase (SOD) (Catalog Number; E-BC-K020, Elabscience Biotechnology Inc. Houston, Texas, United States), catalase (CAT) (Catalog Number; E-BC-K106, Elabscience Biotechnology Inc. Houston, Texas, United States), glutathione peroxidase (GPx) (Catalog Number; E-BC-K096, E-EL-R2491, Elabscience Biotechnology Inc. Houston, Texas, United States), malondialdehyde (MDA) (Catalog Number; E-EL-0060, Elabscience Biotechnology Inc. Houston, Texas, United States), TRIzol reagent (Biobasic BS410A-MA18DR0J, Markham, Ontario, Canada), SYBR Green Master Mix reagents (Thermo Fisher Scientific, Waltham, Massachusetts, United States), cDNA Synthesis Kit (Thermo Scientific RevertAid First-Strand cDNA Synthesis Kit) (Thermo Fisher Scientific, Waltham, Massachusetts, United States), and methanol (HPLC-grade). All standards (purity 95%) for the amino acid analyzer (AAA) were purchased from Sigma-Aldrich (St. Louis, Missouri, United States).

### 2.2 Preparation of solutions

Lead acetate was available in white powder form. The solution of PbAc was prepared in distilled water as it was soluble in water. The dose of PbAc (120 mg/kg) was calculated for individual mice and then dissolved in 0.5 ml of distilled water. Quercetin was available in the form of a coarse powder. It was insoluble in water. For its administration to mice, it was blended in corn oil after calculating the quercetin (50 mg/kg) dose for individual mice. Both PbAc and Que are administered by the oral gavage route to mice.

### 2.3 Experimental design

The study was conducted on 25 Wistar albino mice aged 9 weeks and weighing 30 ± 5 g. During the experimental study, animals were kept in the animal house of Government College University, Faisalabad (GCUF), Pakistan, and acclimatized for 7 days. Mice remained in a well-ventilated and air-conditioned animal room under standard conditions of temperature, 25 ± 2°C, a dark light cycle of 12–12 h, and relative humidity of 50 ± 5%. Mice were fed a pellet diet and would have *ad libitum* access to water. The experimental protocols of this study were followed by the guidelines of “The Institutional Review Board of GCUF” and ethical standards and procedures for research on experimental animals with an authorized reference number Ref. No. GCUF/ERC/32. After acclimatization for 1 week, 25 mice were randomly divided into five groups. The first group received normal saline and was designated as the CON group. The second group was exposed to PbAc at a dose of 120 mg/kg by oral gavage and was designated as the Pb group. The third group was first exposed to PbAc (120 mg/kg) and then treated with quercetin (50 mg/kg) blended in corn oil by oral gavage and designated as the Pb + Q-CRN group. The fourth group was treated with quercetin 50 mg/kg blended in corn oil by oral gavage and was designated as the Q-CRN group. The fifth group received corn oil by oral gavage to investigate any interference in the biochemical profiling due to the administration of corn oil and was designated as the CRN group. The doses of PbAc (120 mg/kg) (Highab et al., 2020) and quercetin (50 mg/kg) (Mert et al., 2019) were adjusted based on the reports available in the literature. Quercetin was not soluble in water, so it was blended in corn oil to administer it as suggested in previous studies (Mert et al., 2019; Mirzakhani et al., 2020). All the treatments were given to mice daily *via* oral gavage for 4 weeks in the morning. After 28 days, the mice were starved overnight. After anesthetizing the mice, they were euthanized by dislocating the cervical bone. This method is ethically acceptable for killing rodents such as mice, rats, squirrels, etc. After euthanizing the mice, whole blood was taken out of their bodies through cardiac puncture for serum and plasma separation for biochemical analysis. The liver was obtained after the dissection of mice for the evaluation of mRNA expression and antioxidant activity. The liver and whole blood were stored in the refrigerator at 4°C. However, the separated plasma and serum were preserved at −20°C and −80°C, respectively, for further analysis.

### 2.4 Estimation of biomarkers of oxidative stress and lipid peroxidation

The liver of mice was obtained and placed in clean polythene bags, washed with ice-cold 0.01 M phosphate buffer saline solution (pH 7.4), which was prepared by dissolving one PBS tablet in 200 ml of distilled water. According to the instructions of the manufacturer, each tablet yields 0.01 M PBS, with a pH of 7.4. This step was repeated thrice to ensure the complete removal of blood and other contaminants present. The liver was then minced, and approximately 1 g was taken out for analysis. The tissue and PBS were homogenized using a manual tissue homogenizer in such a way that the ratio of tissue to PBS was 1:9. To ensure the breakdown of tissues, the resulting homogenized solution was further sonicated, following which the homogenate was centrifuged for 10 min at 10,000 × g and 4°C to separate the debris. The supernatant of liver tissues was collected and used for the estimation of lipid peroxidation (malondialdehyde) and oxidative stress markers (GSH, SOD, CAT, and GPx) using their corresponding ELISA kits according to the manufacturer’s instructions (Irshad et al., 2021).

### 2.5 Evaluation of mRNA expression of impaired lipid and amino metabolism linked gene transcripts

The expression of CPT-I, CPT-II, LCAT, CROT, CACT, and MTR genes was evaluated by qRT-PCR. First, the total RNA pellets were isolated by utilizing the TRIzol reagent (Biobasic BS410A-MA18DR0J) from the liver tissue homogenates that were preserved at 4°C. Then, cDNA was synthesized using the Thermo Scientific RevertAid First-Strand cDNA Synthesis Kit. After the RT reaction, qRT-PCR steps were carried out by filling qRT-PCR plates and making up the volume of each well by 20 μL by using the SYBR Green Master Mix reagents (Thermo Scientific). The primers were designed by Custom DNA Oligos-Eurofins Genomics, and the sequences of the primers are listed in Table 1. The prepared qRT-PCR plate was run on qRT-PCR at thermal cycles of 95°C for 10 min, followed by 40 cycles (denaturation for 15 s at 95°C and annealing for 30 s at 60°C) using a real-time PCR machine. The β-actin gene was selected as an internal reference or housekeeping gene to normalize the gene expression levels. Fold changes in selected genes were calculated by the 2^- ΔΔCt^ method.

**TABLE 1:** List of primers employed in qRT-PCR analysis of targeted genes.

Gene name	Gene symbol	Primer (5′–3′)	Target size (bp)	
β-actin	β-Actin	Forward	CCC​ATC​TAT​GAG​GGT​TAC​GC	150
		Reverse	TTT​AAT​GTC​ACG​CAC​GAT​TTC	
Carnitine palmitoyl transferase I	CPT I	Forward	ATC​CAC​CAT​TCC​ACT​CTG​CT	107
		Reverse	TGT​GCC​TGC​TGT​CCT​TGA​TA	
Carnitine palmitoyl transferase II	CPT II	Forward	CTG​TCC​ACC​AGC​ACT​CTG​AA	111
		Reverse	GCA​ACC​TAT​CCA​GTC​ATC​GT	
Ecithin–cholesterol acyltransferase	LCAT	Forward	CTC​CTT​CTG​GCT​CCT​CAA​TG	171
		Reverse	TCC​TCT​GTC​TTT​CGG​TAG​CAC	
Carnitine O-octanoyltransferase	CROT	Forward	AGA​CGG​AAG​GGA​GAT​GGA​G	168
		Reverse	AAG​ATG​TGA​AGG​TAG​ATG​CTG​CT	
Mitochondrial carnitine/acylcarnitine carrier protein	CACT	Forward	TTC​TCC​ACT​GCT​GCT​CCT​G	100
		Reverse	CCT​GTC​TGC​TCC​CAT​TCA​G	
5-methyltetrahydrofolate-homocysteine methyltransferase	MTR	Forward	GGT​TCG​GTT​GAA​GAA​GAG​GA	112
		Reverse	TAT​TAC​AGC​CCA​GCA​CCA​CA	

### 2.6 Detection and quantification of amino acids by amino acids analyzer

For plasma separation, the whole blood that was taken in an EDTA tube was centrifuged at 2000 rpm for 20 min at 4°C. The separated plasma collected in the Eppendorf tube was stored at −20°C and used for amino acid analysis with the help of the Biochrom Amino Acid Analyzer (AAA).

### 2.6.1 Deproteinization of the blood sample

The deproteinization of plasma was carried out with 5-sulfosalicylic acid by following the manufacturer’s instructions (Kim et al., 2017). The separated plasma was mixed with a 3% 5-sulfosalicylic acid (3 g of 5-SSA in 100 ml of distilled water) solution in a ratio of 1:1. The sulfosalicylic acid leads to the precipitation of proteins in plasma. The contents of the tube were mixed immediately and then allowed to stand for 30 min at 4°C, following which, the Eppendorf tube was then centrifuged at 10,000 × g at 4°C for 5 min until a clear supernatant was obtained.

### 2.7 Qualitative analysis of lipid metabolomes by MS/MS

**FIGURE 2:**
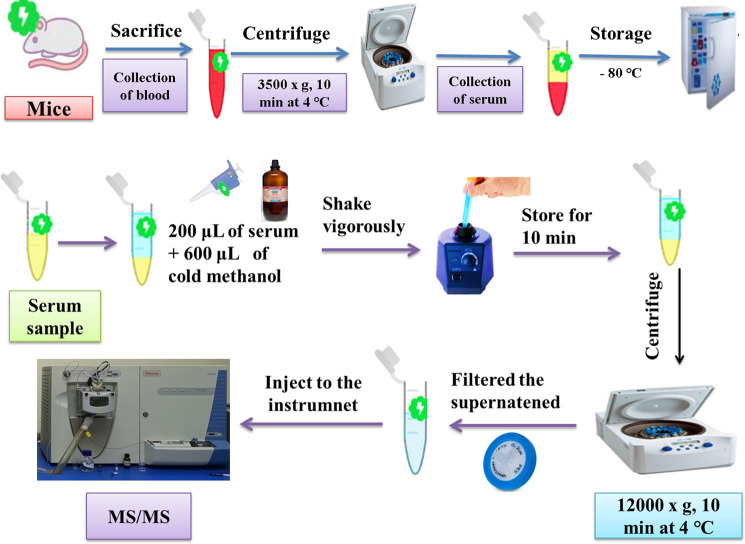
Schematic representation of serum separation and sample pretreatment for metabolite acquisition by MS/MS.

The whole blood was centrifuged at 3,500 × g for 10 min at 4°C, and the serum was collected. The serum was then stored at −80°C before the metabolomics analysis. The serum sample was pretreated by adding 600 μL of cold methanol to 200 μL of the serum and shaking vigorously. The mixture was stored for 10 min, followed by centrifugation at 12,000 × g for 10 min at 4°C. The supernatant was then filtered through 0.22-μm polytetrafluoroethylene polymer (PTFE) filters before injection into an ion trap mass spectrometer for tandem mass spectrometry (MS/MS). Serum separation and sample pretreatment for metabolite acquisition by ESI-MS are shown in Figure 2. The general conditions for sample analysis by MS/MS are shown in Table 2. The qualitative analysis of serum metabolomes by MS/MS was performed at the National Institute of Biotechnology and Genetic Engineering (NIBGE), Faisalabad, Pakistan.

**TABLE 2:** General conditions for sample analysis by MS/MS.

Instrument	Linear ion trap mass spectrometer model; LTQ XL (Thermo Electron Scientific, United States) equipped with electrospray ionization source
Solvent	Methanol
Mode of injection	Direct insertion method
Flow rate	9.8 μL/min
Mode of ionization	Both negative and positive scan ion modes
Capillary voltage	4.7 kV
Capillary temperature	278°C
Sheath gas flow rate	17 units
Auxiliary gas flow rate	Six units
Scanning mass range	50–2000°m*/z*
Fragmentation (MS/MS)	Various peaks were selected for fragmentation, using collision-induced dissociation (CID) energy ranging from 20–30
Software	Xcalibur 2.0.7

### 2.8 Statistical analysis

The results were estimated as mean ± SD. One-way ANOVA was used to determine the significant difference between the groups when the value of probability was considered as (*p* < 0.05) by using GraphPad Prism 5 (GraphPad Software Inc, La-Joya, CA, United States). The graphical data were represented as mean ± SD.

## 3 Results

### 3.1 Effect of PbAc on biomarkers of oxidative stress and lipid peroxidation

**FIGURE 3:**
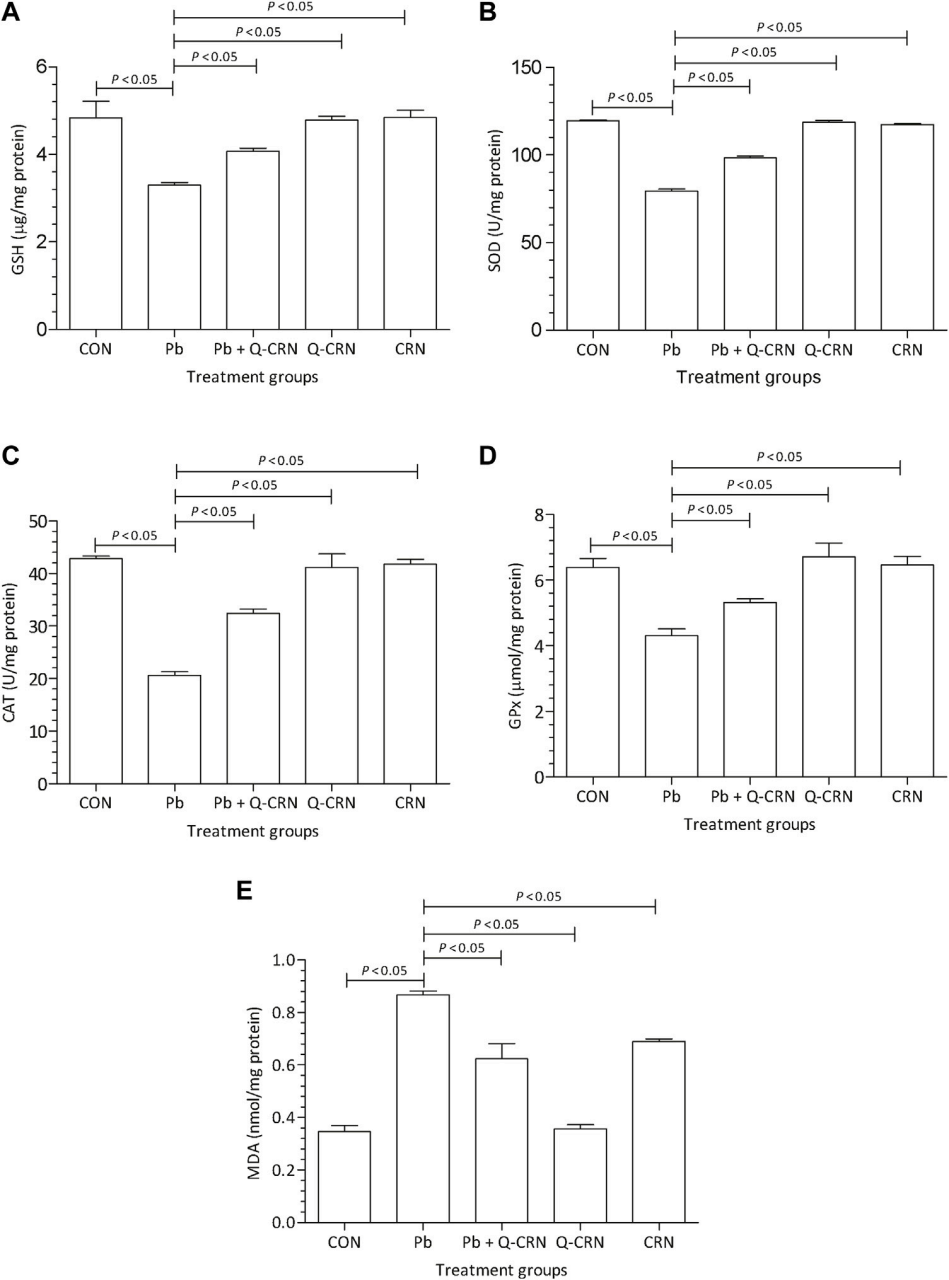
Effect of intoxication of Pb on the liver **(A)** GSH, **(B)** SOD, **(C)** CAT, **(D)** GPx, and **(E)** MDA and the ameliorative effect of Que. The levels of GSH, SOD, CAT, GPx, and MDA were measured at the end of the experiment in the hepatic tissue homogenate. The level of significance was set at *p* < 0.05, and the data were analyzed using one-way ANOVA followed by Tukey’s test to compare all pairs of columns. The results were expressed as mean ± SD. Abbreviations: GSH: glutathione; SOD: superoxide dismutase; CAT: catalase; GPx: glutathione peroxidase; MDA: malondialdehyde; CON: control group; Pb: lead group; Pb + Q-CRN: lead and quercetin blended in corn oil group; Q-CRN: quercetin blended in corn oil group; CRN: corn oil group; ANOVA: analysis of variance.

The findings of our study represented a significant (*p* < 0.05) decrease in SOD, GSH, GPx, and CAT levels after the intoxication of mice with PbAc as compared to the CON group (Figure 3). We also determined a significant (*p* < 0.05) elevation in MDA levels after the intoxication of mice with PbAc as compared to the CON group (Figure 3E). The most effective method for this restoration of the antioxidant system is the use of naturally occurring antioxidants. In the present study, the natural flavonoid quercetin was used. In the Pb + Q-CRN group, the findings showed that when Pb-intoxicated mice were treated with quercetin blended in corn oil, the levels of all the above-mentioned biomarkers were restored significantly (*p* < 0.05) as compared to those of the PbAc exposure group. Thus, our study confirmed the tendency of PbAc to cause oxidative stress and lipid peroxidation in mice and the ameliorative effect of the bioflavonoid quercetin in treating oxidative stress and lipid peroxidation. The level of MDA was also significantly increased in the corn oil group, thus confirming the effect of corn oil on lipid peroxidation.

### 3.2 Effect of PbAc on gene expression of impaired lipid and amino metabolism

**FIGURE 4:**
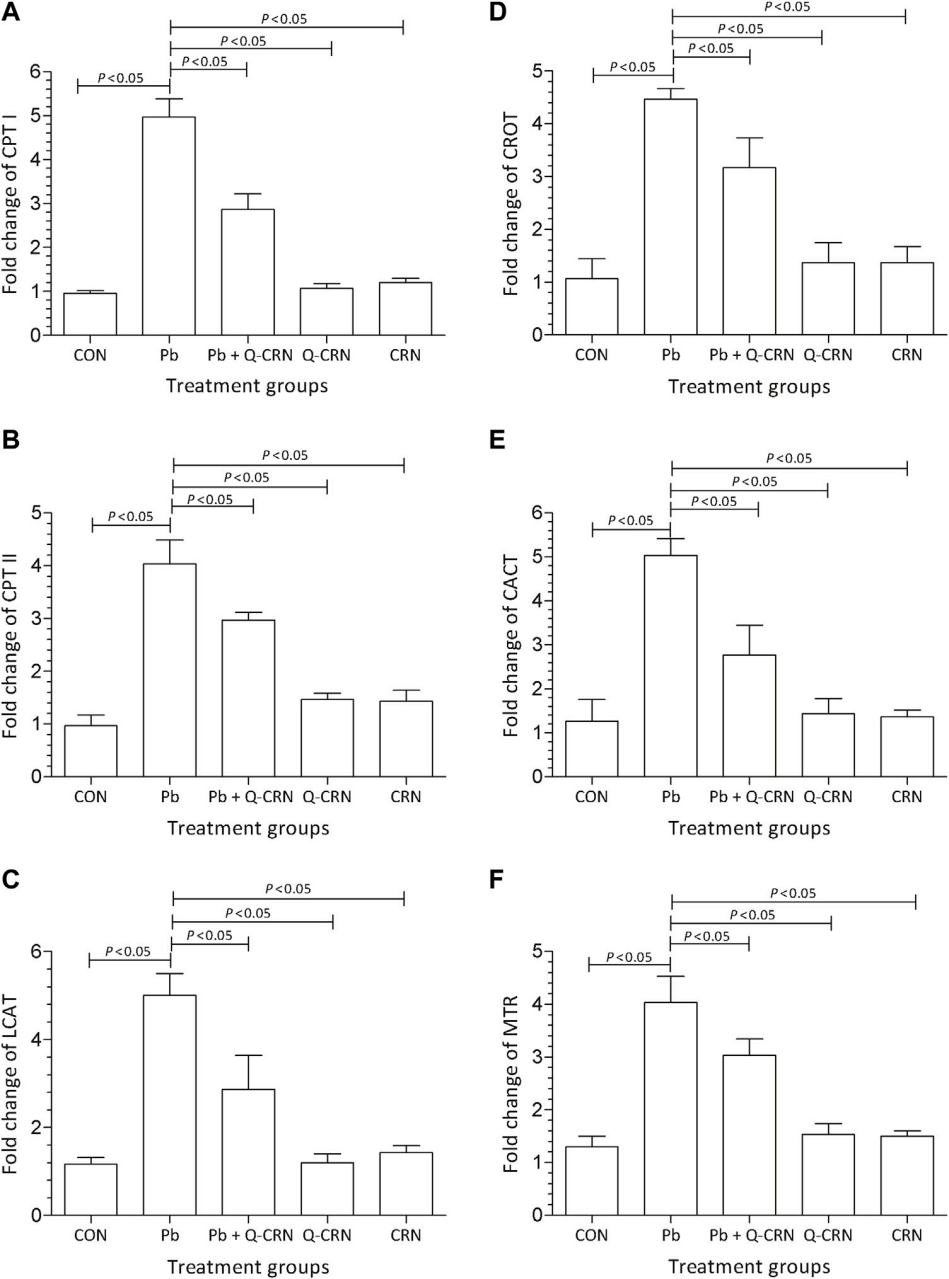
Bar diagram represents the effect of PbAc on mRNA expression of the **(A)** CPT I, **(B)** CPT II, **(C)** LCAT, **(D)** CROT, **I** CACT, and **(F)** MTR genes in different mice groups, i.e., CON, Pb, Pb + Q-CRN, Q-CRN, and CRN groups, respectively. The quantitative analysis was carried out at the end of the experiment of 28 days. The significance level was set at (*p* < 0.05) by using one-way ANOVA followed by Tukey’s test to compare all pairs of columns. Each error bar represents the mean ± SD. Abbreviations: CPT I carnitine palmitoyltransferase I; CPT II: carnitine palmitoyltransferase II; LCAT: lecithin–cholesterol acyltransferase; CROT: carnitine O-octanoyltransferase; CACT: mitochondrial carnitine/acylcarnitine carrier protein; MTR: 5-methyltetrahydrofolate-homocysteine methyltransferase; CON: control group; Pb: lead group; Pb + Q-CRN: lead and quercetin blended in corn oil group; Q-CRN: quercetin blended in corn oil group; CRN: corn oil group; ANOVA: analysis of variance.

We observed that when the diseased group of mice exposed to PbAc was compared with the CON group for determining the expression of carnitine palmitoyltransferase-I (CPT-I), carnitine palmitoyltransferase-II (CPT-II), lecithin–cholesterol acyltransferase (LCAT), carnitine O-octanoyltransferase (CROT), mitochondrial carnitine/acylcarnitine carrier protein (CACT), and 5-methyltetrahydrofolate-homocysteine methyltransferase (MTR) genes, it was found that a significant (*p* < 0.05) upregulation in the expression of these genes in the Pb exposed group was observed as compared to that of the CON group (Figure 4). However, the findings confirmed that the overexpression of these genes was reduced significantly (*p* < 0.05) in the Pb + Q-CRN group as compared to the diseased Pb group. This also confirmed the beneficial effect of quercetin against Pb toxicity in mice.

### 3.3 Effect of PbAc on the area percent of amino acids in mouse plasma

**FIGURE 5:**
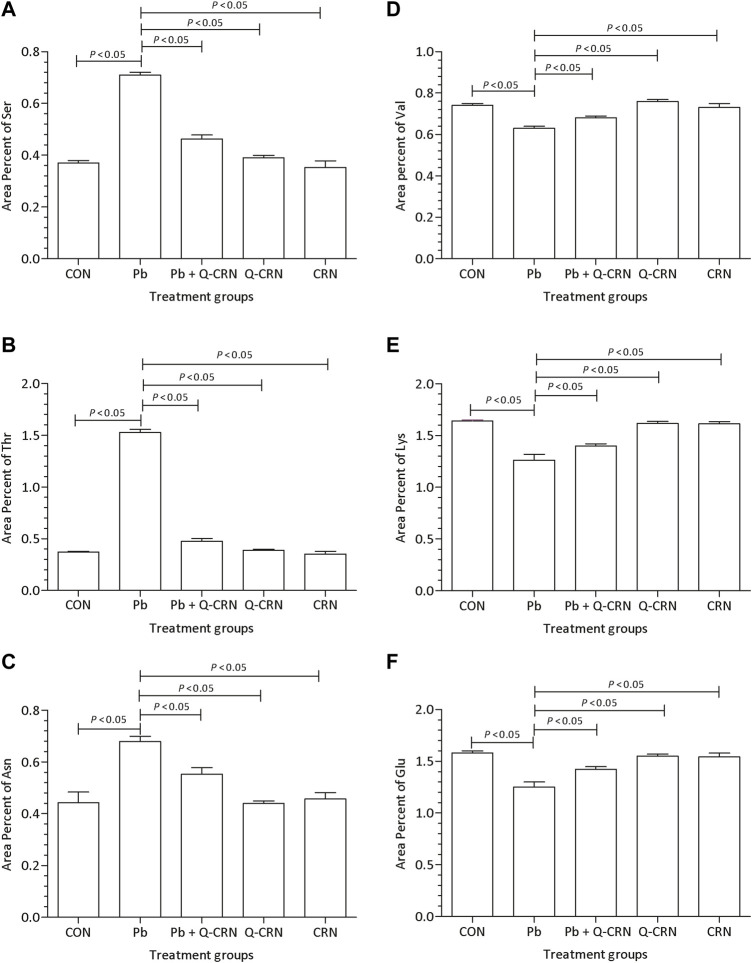
Effect of intoxication of PbAc on area percent of **(A)** serine, **(B)** threonine, **(C)** asparagine, **(D)** valine, I**(E)** lysine, and **(F)** glutamic acid in the plasma of different mice groups, i.e., CON, Pb, Pb + Q-CRN, Q-CRN, and CRN groups, respectively. The quantitative analysis was carried out at the end of the experiment of 28 days. The significance level was set at (*p* < 0.05) by using one-way ANOVA followed by Tukey’s test to compare all pairs of columns. Each error bar represents the mean ± SD. Abbreviations: Ser: serine; Thr: threonine; Asn: asparagine; Val: valine; Lys: lysine; Glu: glutamic acid; CON: control group; Pb: lead group; Pb + Q-CRN: lead and quercetin blended in corn oil group; Q-CRN: quercetin blended in corn oil group; CRN: corn oil group ANOVA: analysis of variance.

When the diseased group of mice exposed to PbAc was compared with the CON group for determining the difference in the area percent of serine, threonine, and asparagine amino acids, it was found that Pb exposure significantly (*p* < 0.05) increased the area percent of serine (Figure 5A), threonine (Figure 5B), and asparagine amino acids (Figure 5C) as compared to the CON group. However, the levels of valine (Figure 5D), lysine (Figure 5F), and glutamic acid (Figure 5F) amino acids were significantly (*p* < 0.05) reduced in the Pb group. However, the level of the above-mentioned amino acids was restored in the Pb + Q-CRN group when compared to the diseased Pb group, showing the beneficial effect of quercetin against Pb toxicity.

### 3.4 Effect of PbAc on serum lipid metabolomes

In this study, the qualitative analysis of serum samples of two important groups was performed. The first was the diseased group that was exposed to Pb only, and the second was the treated group, to whom, after Pb exposure, the mice were treated with quercetin blended in corn oil. Four important lipid metabolites were identified from both the positive and negative modes of the full MS/MS scan. The compounds detected in both the Pb and Pb + Q-CRN groups, their molecular formula, molecular weight, precursor m/z, and product ion m/z are elaborated in Table 3.

**TABLE 3:** Compounds detected and confirmed in the serum sample of mice.

Compound	Molecular formula	Molecular weight	Polarity	Precursor ion (m/z)	Product ion (m/z)	Detected in	
						Pb group	Pb + Q-CRN group
Carnitine	C_7_H_16_NO_3_	162	Negative	160.75	142.9, 117, and 103.08	✓	✓
Sphinganine	C_18_H_39_NO_2_	302	Positive	303	285, 190.3, 176.3, 150, 119.92, and 106	✓	✓
Phytosphingosine	C_18_H_39_NO_3_	317	Positive	318	300 and 256	✓	✓
Lysophosphatidylcholine	C_26_H_54_NO_7_P	523	Positive	546	487, 341, 404, and 443	✓	✓

#### 3.4.1 L-Carnitine

**FIGURE 6:**
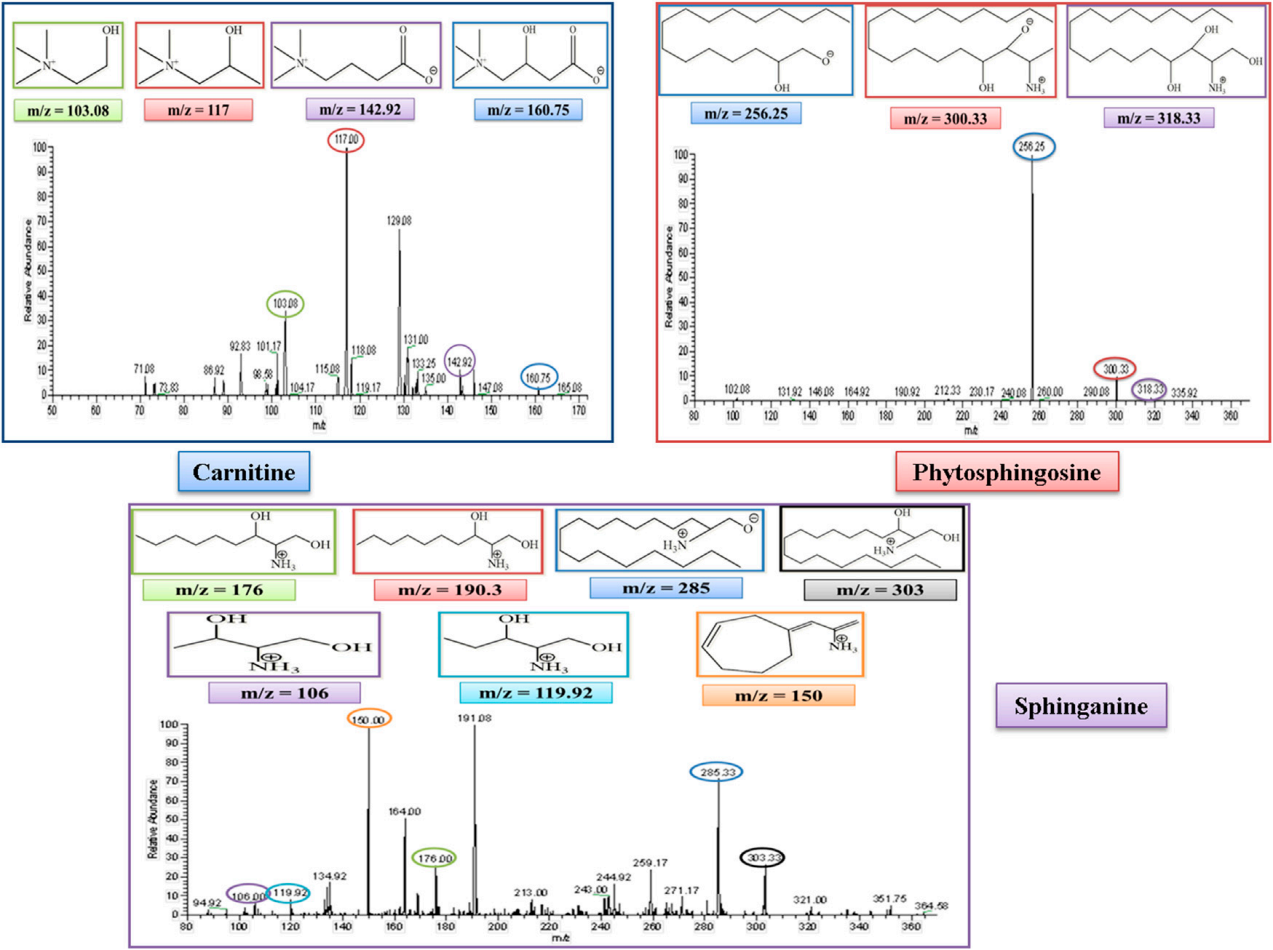
MS/MS spectrum of L-carnitine, phytosphingosine, and sphinganine in negative and positive ion mode, respectively. Each m/z peak is circled by a color similar to the color of the border of its structure.

L-Carnitine belongs to the fatty acid β-oxidation which is a sub-pathway of lipid metabolism. The molecular weight of carnitine is 162. In the MS/MS spectrum, it showed its peak at 160.75, confirming its presence in the serum sample in the negative ion mode of the MS/MS spectrum. The peak of carnitine is shown on the extreme right side of the graph. After the removal of a water molecule, the carboxylic group and ethanoic acid carnitine showed their fragment peaks at m/z of 142.9, 117, and 103.08, respectively, as represented in Figure 6.

#### 3.4.2 Phytosphingosine

Phytosphingosine belongs to the sphingolipid metabolism, which is a sub-pathway of lipid metabolism. The molecular weight of phytosphingosine is 317. In the MS/MS spectrum, we observed a peak at 318, which confirmed its presence in the serum sample in the positive ion mode of the MS/MS spectrum. After the removal of a single water molecule and 2-hydroxyethyl amine, phytosphingosine showed its fragment peak at an m/z of 300 and 256, respectively, as represented in Figure 6.

#### 3.4.3 Sphinganine

Sphinganine belongs to sphingolipid metabolism, which is a sub-pathway of lipid metabolism. The molecular weight of sphinganine is 302. In the MS/MS spectrum, the peak was observed at 303, which confirmed its presence in the serum sample in the positive ion mode of the MS/MS spectrum. After the removal of a water molecule, the peak was observed at an m/z of 285. The MS spectrum also showed sphinganine fragments after the removal of the alkyl chain of 8, 9, 13, and 14 carbons and showed its peak at m/z of 190.3, 176.3, 119.92, and 106, respectively. The peak at 190.3 overlapped with another peak at an m/z of 191.08, which was thought to be due to the presence of an isotope. It also showed a fragment after the rearrangement at an m/z of 150, as represented in Figure 6.

#### 3.4.4 Lysophosphatidylcholine

**FIGURE 7:**
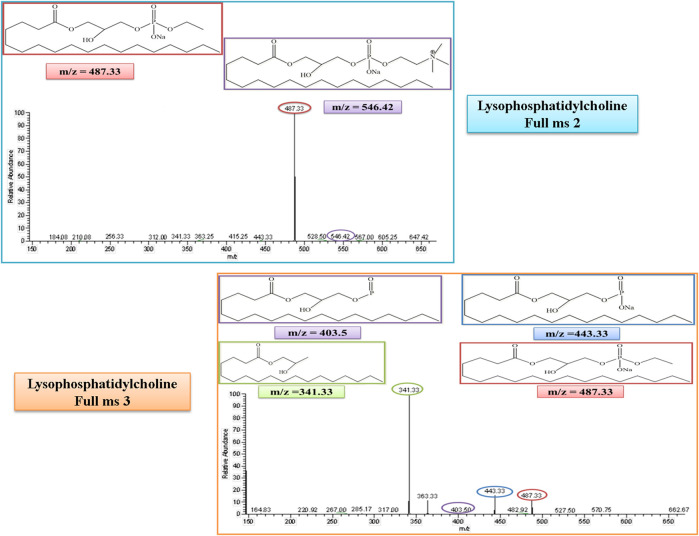
Full ms 2 and full ms 3 spectra of Lysophosphatidylcholine in positive ion mode. Each m/z peak is circled by a color similar to the color of the border of its structure.

Lysophosphatidylcholine (LysoPC) belongs to the lysolipid metabolism, which is a sub-pathway of lipid metabolism. The molecular weight of lysophosphatidylcholine (LysoPC) is 523. In the MS/MS spectrum, its peak was observed at 546, which confirmed its presence in the serum sample in the positive ion mode of the MS/MS spectrum after the addition of a sodium molecule. After the removal of a trimethylammonium ion, the rest of the fragment molecule showed its peak at m/z of 487, as represented in Figure 7. This peak was further fragmented into full ms 3 as shown in Figure 7, and the product ion peaks of LysoPC appear at the m/z of 341, 404, and 443.

## 4 Discussion

Exposure to Pb is widely spreading, and it has far-reaching negative effects on the physiological mechanism. To date, only a few studies have reported the metabolomics analysis of Pb exposure. In these studies, plasma and urinary metabolites were determined in the participants after occupational and residential Pb exposure (Dudka et al., 2014; Eguchi et al., 2018; Kelly et al., 2020). The current study exhibited the potentially harmful effect of Pb on oxidative stress, gene expression, and lipid and amino acid metabolism by inducing Pb intoxication with the help of PbAc, and then the ameliorative effect of plant-derived bioflavonoid compound quercetin against Pb toxicity has also been demonstrated in the experimental male Wistar albino mice.

The finding of this study demonstrated that the level of SOD, GSH, GPx, and CAT was decreased in the Pb-intoxicated mice group and was responsible for oxidative stress in mice, as the same has also been reported in some previous studies (Wang et al., 2013; Javorac et al., 2021). The levels of SOD, GSH, GPx, and CAT were increased in the Pb + Q-CRN group because of the antioxidant activity of bioflavonoid quercetin as already reported in previous studies (Crown et al., 2019; Akinmoladun et al., 2020). The administration of corn oil to mice did not have any effect on SOD levels (Haggag Mel et al., 2014).

Blood Pb toxicity is also responsible for disturbing the heme biosynthetic pathway. In the blood, Pb inhibits δ-aminolevulinic acid dehydratase (ALAD), and thus the concentration of aminolevulinic acid (ALA) is increased in the blood, which leads to oxidative stress in the body (Qader et al., 2021). It was presumed that the high level of ALA was responsible for increasing the level of MDA in Pb-intoxicated mice (Wang et al., 2013; Kasperczyk et al., 2015; Javorac et al., 2021). However, the level of MDA was restored in the Pb + Q-CRN group because of the antioxidant activity of bioflavonoid quercetin, as also confirmed in previous studies (Crown et al., 2019; Akinmoladun et al., 2020). There was also a significant increase in the level of MDA in the CRN group because corn oil was responsible for increasing lipid peroxidation in the mice, as reported in a previous study (Haggag Mel et al., 2014).

**FIGURE 8:**
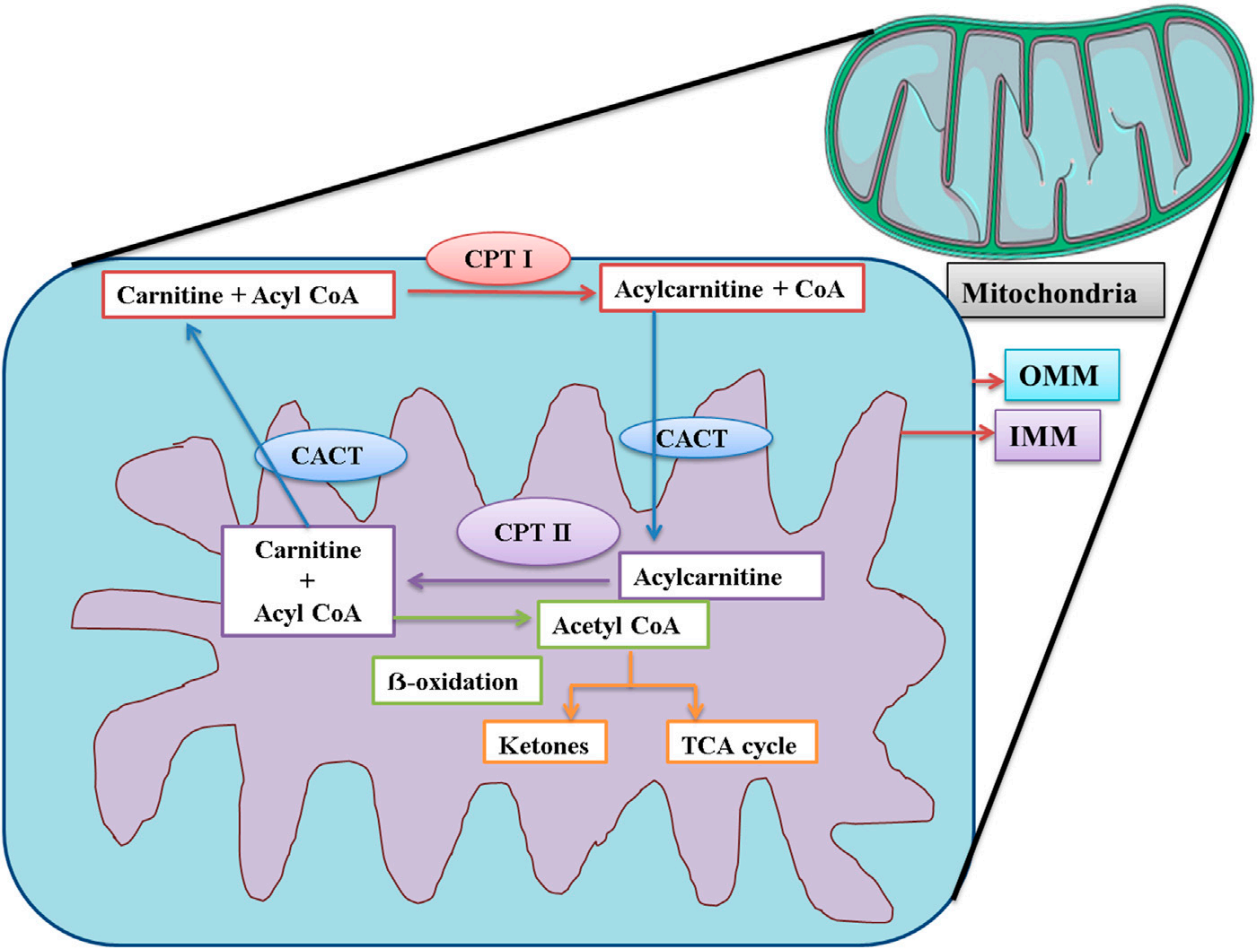
Schematic representation of the functions of CPT I, CPT II, and CACT in mitochondria. CPT I is present at OMM. CPT I attaches a long-chain fatty acid, acyl CoA (palmitoyl-CoA and octadecenyl-CoA) to carnitine, and the resultant acylcarnitine is transported to the mitochondrial matrix by CACT. CACT is located at the IMM. Once inside the mitochondria, acylcarnitine is again converted to free carnitine and Acyl CoA. This free carnitine is again transported by CACT. The acyl CoA undergoes β-oxidation and is converted to acetyl CoA. The acetyl CoA can be converted into ketone bodies or enter the TCA cycle. CPT I: carnitine palmitoyltransferase I; CPT II: carnitine palmitoyltransferase II; CACT: mitochondrial carnitine/acylcarnitine carrier protein; OMM: outer mitochondrial membrane; IMM: inner mitochondrial membrane.

**FIGURE 9:**
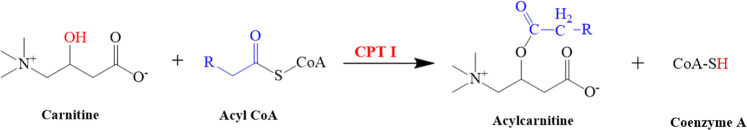
Illustrated the conversion of carnitine into acylcarnitine. This reaction is catalyzed by CPT I.

**FIGURE 10:**
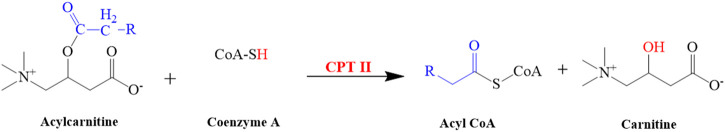
Illustrated the conversion of acylcarnitine into carnitine. This reaction is catalyzed by CPT II.

CPT-I is present at the outer mitochondrial membrane (Figure 8), and CPT-II is present on the inner mitochondrial membrane (Figure 8), both of which are responsible for catalyzing the reactions as described in Figures 9, 10 respectively (Wang et al., 2011; Joshi and Zierz, 2020).

CACT is located on the inner mitochondrial membrane. Its function is the translocation of free carnitine and acylcarnitine molecule as shown in Figure 8 (Wang et al., 2011; Joshi and Zierz, 2020). The expression of CPT-I, CPT-II, and CACT was upregulated, strongly suggesting that the level of carnitine would also be elevated in the mice serum. The findings of our study were supported by those of a previous study that confirmed that Pb exposure was responsible for DNA hypermethylation, thus causing the upregulation of genes (Sun et al., 2017). The level of carnitine and expression of these genes were also upregulated in the findings of some other studies (Shen et al., 2013; García-Sevillano et al., 2014a; Wang et al., 2015).

**FIGURE 11:**
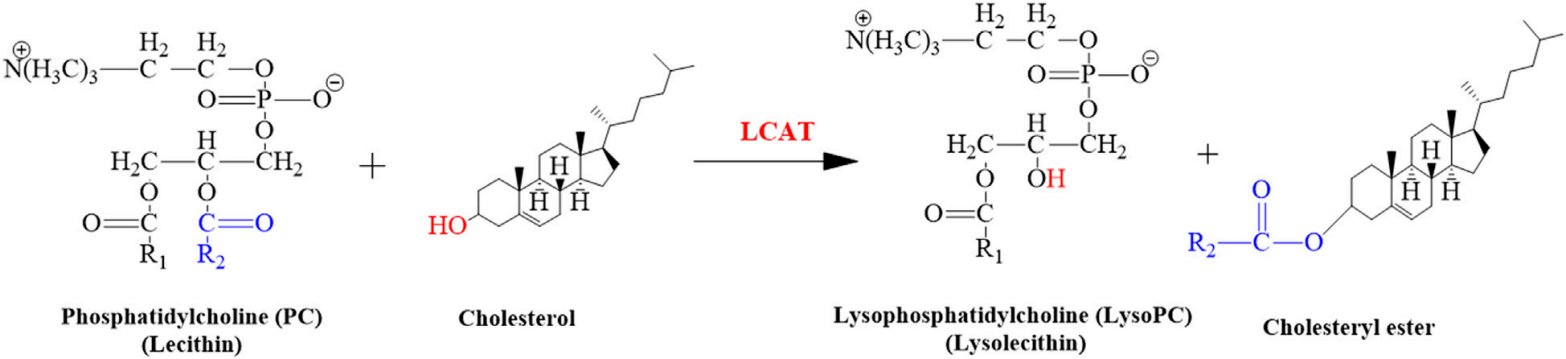
Illustrated the conversion of cholesterol into cholesteryl ester. This reaction is catalyzed by LCAT.

The LCAT gene is responsible for giving instructions for the synthesis of the LCAT enzyme that removes cholesterol from the tissues and blood of the body and synthesizes the LysoPC in plasma as shown in the following reaction (Figure 11). The expression of the hepatic LCAT gene was upregulated after lead exposure (Sun et al., 2017). The findings of our study suggested that the level of LysoPC would also be increased in serum samples of mice after Pb toxicity, as it was also increased after arsenic exposure in rat’s serum by disrupting the transformation of LysoPC (García-Sevillano et al., 2013; García-Sevillano et al., 2014a; García-Sevillano et al., 2014b; Wang et al., 2015).

Sphinganine and phytosphingosine were also detected in the serum samples of mice. In the lipid metabolic pathway, sphinganine and phytosphingosine are part of sphingolipid metabolism, which is related to the homeostasis of phosphatidylcholine (PC) (Quinville et al., 2021). We hypothesized that Pb exposure increases the level of sphinganine and phytosphingosine by increasing sphingolipid metabolism (Liu et al., 2021). The results of another study concluded that Pb exposure was responsible for decreasing the level of sphinganine and sphingosine in blood plasma (Kelly et al., 2020). In another study, it was confirmed that exposure to arsenic was responsible for elevating the levels of sphinganine and phytosphingosine (Wang et al., 2015).

CROT encodes a member of the carnitine/choline acyltransferase family. CROT is present in peroxisomes and is involved in transesterification reactions. CROT plays an important role in fatty acid beta-oxidation and lipid metabolism (Okui et al., 2021). The expression of CROT was also upregulated in this study, which was also confirmed by some other studies (Ren et al., 2011; Carlson and Van Beneden, 2014; Sun et al., 2017).

**FIGURE 12:**
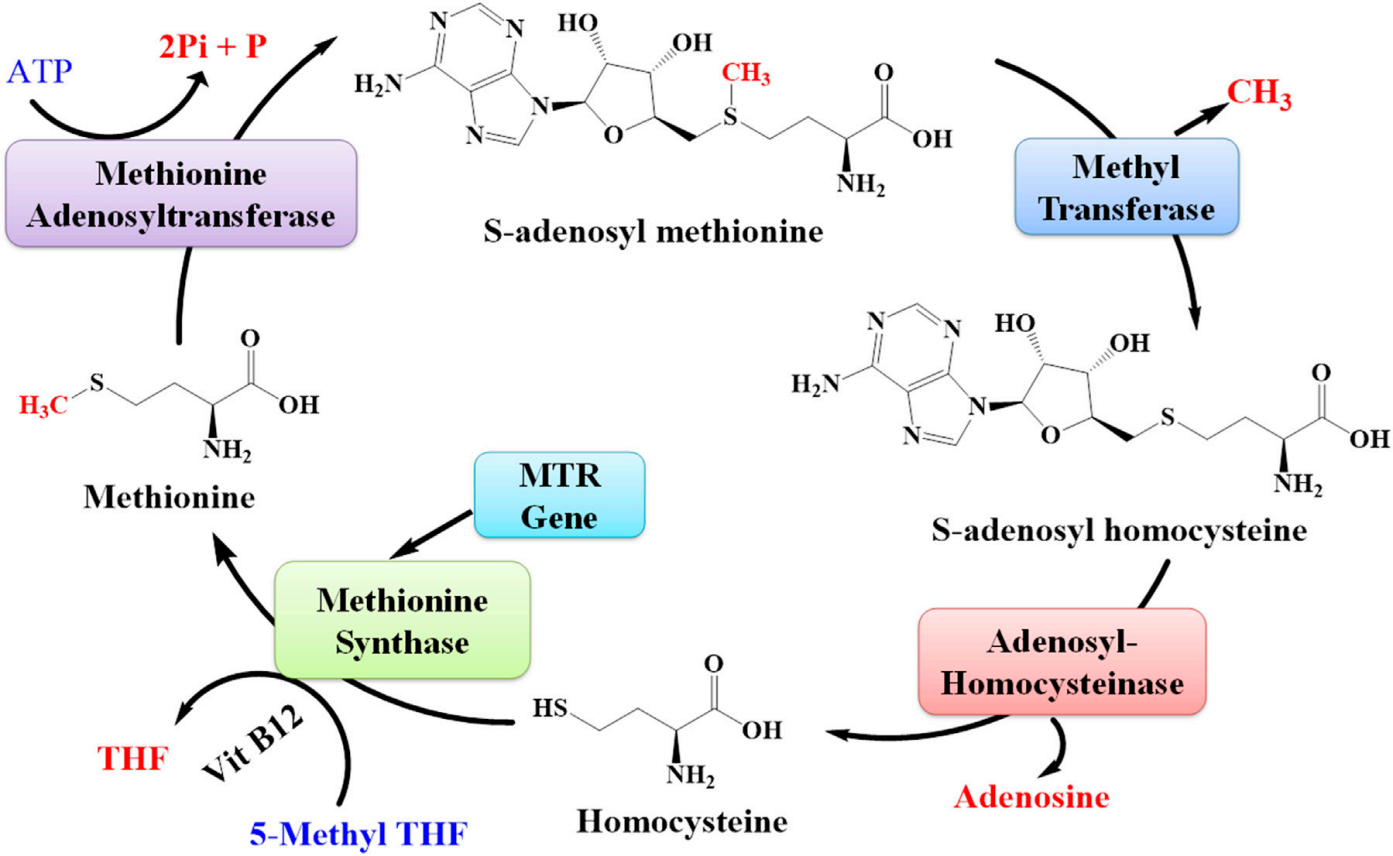
Pathway for the synthesis of methionine from S-adenosyl methionine. The MTR gene is responsible for synthesizing the enzyme methionine synthase that plays an important role in the synthesis of methionine amino acids.

MTR is the gene that provides directions for the synthesis of the methionine synthase enzyme. Methionine synthase is involved in the synthesis of methionine (Figure 12). The living body utilizes this methionine in the synthesis of other proteins and important compounds (Froese et al., 2019). The expression of the MTR gene was also upregulated due to lead toxicity (Wang et al., 2015; Sun et al., 2017).

The amino acids belonging to several interconnected amino acid metabolic pathways that were significant in our study were valine, serine, threonine, lysine, glutamic acid, and asparagine. Some of these amino acids and their metabolic pathways were significantly dysregulated by Pb exposure, as found in some previous studies. The exact mechanism of lead toxicity on amino acid metabolism still needs to be addressed. However, a hypothesis is provided that the expected mechanism of Pb toxicity is the chemical affinity of Pb with non-protein and protein thiols and the Fenton mechanism of generating free radicals and compromising the antioxidant system in the living system (Rubino, 2015). However, another study also found that the dysregulation in the amino acid metabolic pathway is the common response in both animal and human studies in response to different toxins. They also suggest that it is an overall reaction to various toxicants rather than a particular reaction to a particular toxicant.

**FIGURE 13:**
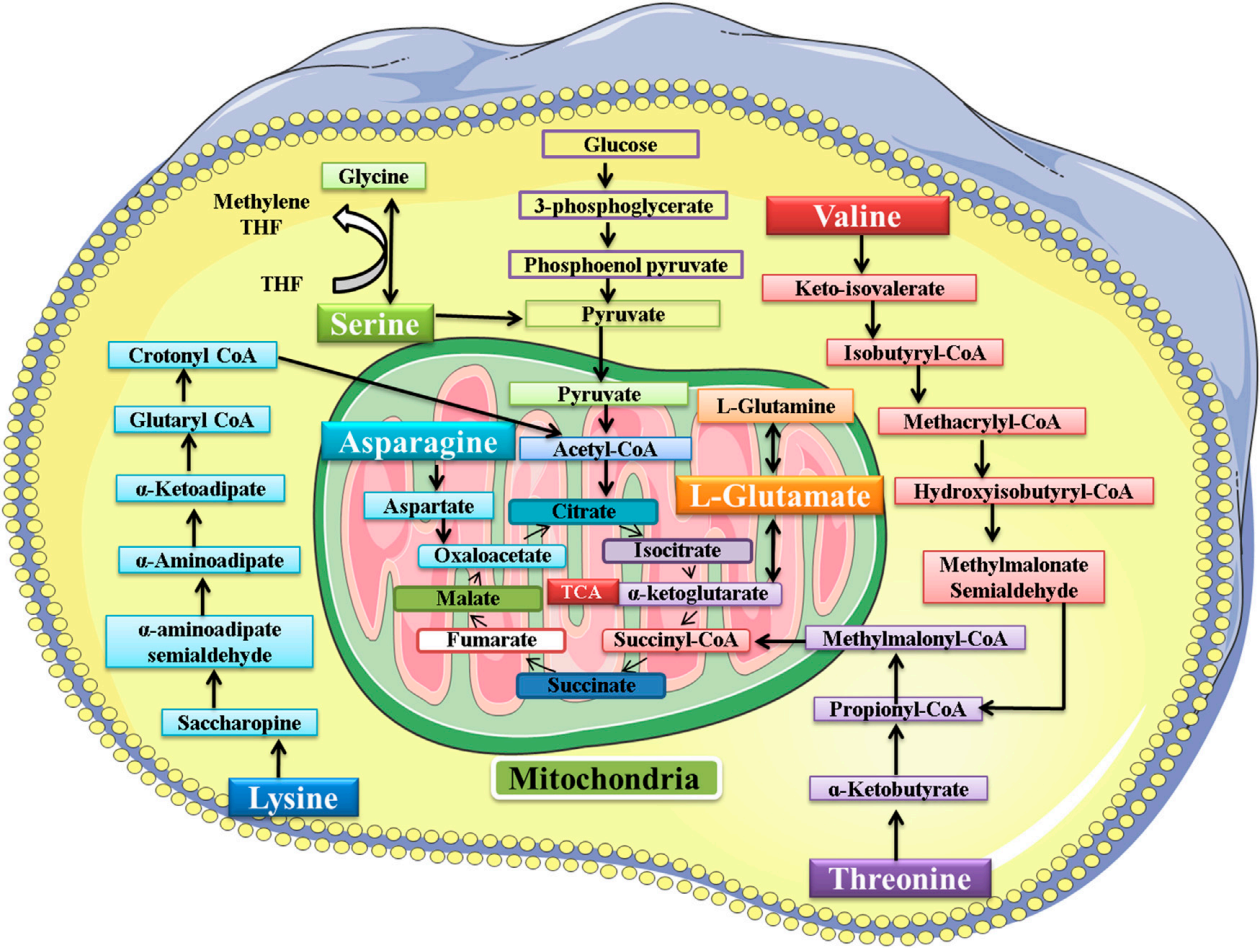
Metabolic pathway of valine, serine, threonine, lysine, glutamic acid, and asparagine. After metabolism, the final product of these amino acids enters the tricarboxylic acid (TCA) cycle.

The metabolic pathway of these six amino acids is shown in Figure 13. This study found that the exposure to Pb was responsible for decrease in the level of valine (Kelly et al., 2020). The serum valine level was significantly decreased after exposure to arsenic, which also supported our findings (Wang et al., 2015). The level of lysine had a negative correlation with the Pb level in plasma (Kelly et al., 2020; Li et al., 2020). Exposure to arsenic was also responsible for decreasing the level of lysine in plasma (Martin et al., 2015). In our study, the results signified that the level of glutamic acid had a negative correlation with the Pb level in the plasma of mice (Kelly et al., 2020; Li et al., 2020). In another study, the effect of methylmercury (MeHg) on metabolomics was also determined, and the level of glutamic acid was also decreased in this study (Reardon et al., 2019).

The Pb toxicity increased the level of serine in the plasma of mice (Kelly et al., 2020). The serum serine level was significantly increased after exposure to arsenic, which also supported our findings (Wang et al., 2015). In another study, the effect of methylmercury (MeHg) on metabolomics was also determined, and MeHg was responsible for increasing the level of serine in Sprague−Dawley rats (Reardon et al., 2019). Pb toxicity was responsible for increasing the level of threonine in the plasma of the mice. The effect of Pb toxicity on threonine level was not explored previously; however, in another study, MeHg was found responsible for increasing the level of threonine (Reardon et al., 2019). In our study, the results showed that the level of asparagine had a positive correlation with the Pb level in the plasma of mice (Kelly et al., 2020).

The increase or decrease in the level of amino acids leads to an impairment in the metabolism of the respective amino acid. When there is impairment in serine metabolism, then the activation of T-cells and proliferation of several other immune cells is compromised (Zhao et al., 2020). The increase in the level of threonine leads to impairment in protein synthesis, intestinal health and function, lipid metabolism, and embryonic stem cell proliferation and differentiation (Kliegman et al., 2020). Asparagine metabolism was also disrupted due to Pb exposure, and thus its functions of synthesizing proteins and neurotransmitters and detoxification of ammonia were also compromised (Lomelino et al., 2017). Valine is responsible for synthesizing proteins and serves as the power supply in the completion of various reactions. Pb toxicity impaired its metabolism and ultimately the synthesis of various proteins (Wolfe, 2017). Lysine is also responsible for synthesizing proteins, peptides, and non-peptide molecules. The impairment in its metabolism compromised its functions in the body (Liao et al., 2015). Glutamic acid also played its role in the body by synthesizing proteins, and the impairment in its metabolism affected the protein synthesis in the body (Kliegman et al., 2020).

## 5 Conclusion

This study investigated the toxic influences of Pb on lipid and amino acid metabolism and oxidative stress, along with the beneficial effect of quercetin. The findings indicated that Pb causes impairment in lipid and amino acid metabolism and increased oxidative stress in the liver. Although the exact mechanism of Pb toxicity against lipid and amino acid metabolism is still unknown, however, it is speculated that the impairment in lipid and amino acid metabolism was a general response of a living system toward different toxicants. However, a hypothesis is provided that the expected mechanism of Pb toxicity is the chemical affinity of Pb with non-protein and protein thiols and the Fenton mechanism of generating free radicals and compromising the antioxidant system in the living system. It is also hypothesized that Pb induces its toxic effect on the body through oxidative stress. Hence, the coadministration of an anti-oxidant bioflavonoid quercetin with Pb helped in reducing Pb toxicity because of its strong antioxidant activity.

### Future perspective

To understand the exact mechanism of Pb toxicity in imparting the lipid and amino acid metabolism, more investigations are needed. This study focuses only on the effect of Pb toxicity on blood metabolomics in mice; thus, there is a need to perform similar studies on urinary metabolomics and humans also. The duration of this study was 28 days; thus, further studies are required to study the effect of long-term Pb exposure on metabolomics and thus confirm its exact pathway of toxicity in impairment of metabolism.

## Data Availability

All data generated and/or analyzed during this study are included in this article.

## Abbreviations

AAA, amino acid analyzer; CACT, mitochondrial carnitine/acylcarnitine carrier protein; CAT, catalase; CON, control group; CPT I, carnitine palmitoyltransferase I; CPT II; carnitine palmitoyltransferase II; CRN, corn oil group; CROT, carnitine O-octanoyltransferase; ELISA, enzyme-linked immunosorbent assay; EDTA, ethylenediamine tetraacetic acid; GPx, glutathione peroxidase; GSH, glutathione; LCAT, lecithin–cholesterol acyltransferase; MDA, malondialdehyde; mRNA, messenger ribonucleic acid; MTR, 5-methyltetrahydrofolate-homocysteine methyltransferase; Pb, lead group; Pb + Q-CRN, lead and quercetin blended in corn oil group; PbAc, lead acetate; PBS, phosphate-buffered saline; PTFE, polytetrafluoroethylene polymer; Q-CRN, quercetin blended in corn oil group; qRT-PCR, real-time quantitative reverse transcription PCR; Que, quercetin; ROS, reactive oxygen species; rpm, revolution per minute; SOD, superoxide dismutase; SSA, sulfosalicylic acid.
